# Initiative to Reduce Antibiotic Exposure of Asymptomatic Infants Born to Mothers with Intraamniotic Infection

**DOI:** 10.1097/pq9.0000000000000480

**Published:** 2021-09-24

**Authors:** Katherine J. Weiss, Richard S. Song, Nikole M. DeVries, Amy L. McLean, Laurel B. Moyer

**Affiliations:** From the * Division of Neonatology, Department of Pediatrics, University of California, San Diego, La Jolla, Calif.; † Rady Children’s Hospital, San Diego, Calif.; ‡ Rancho Springs Medical Center, Southwest Healthcare System, Murrieta, Calif.

## Abstract

Infants born to mothers with intraamniotic infection (IAI) received antibiotic treatment per the Centers for Disease Control and Prevention and American Academy of Pediatrics guidelines in our neonatal intensive care unit (NICU) for early-onset bacterial sepsis evaluation. We conducted a quality improvement project to decrease antibiotic use and NICU admission in infants born to mothers with IAI.

**Methods:**

We aimed to decrease the antibiotic exposure for asymptomatic infants born to mothers with IAI from 100% to 20% in 6 months. We obtained baseline data on these infants from January 2018 to January 2019, with the intervention starting in February 2019. A new standardized guideline to clinically monitor and follow laboratories on asymptomatic infants in couplet care was created with a multidisciplinary team’s help and implemented after provider education. The team reviewed data monthly and used PDSA cycles to make necessary changes, including updating order sets, more educational handouts, and real-time coaching to both nurses and physicians.

**Results:**

There was a dramatic decline (93%–0%) in antibiotic exposure and NICU admission after implementing this guideline. There was also a decrease in IAI diagnosis. There were no readmissions of infants for infection within 30 days of discharge, and there were no positive blood cultures.

**Conclusions:**

Implementing best antibiotic stewardship practices through a standardized guideline, testing, implementation of processes, and education by a multidisciplinary team limited the antibiotic exposure and NICU admissions for infants born to mothers with IAI with no known increase in readmissions.

## INTRODUCTION

Intraamniotic infection (IAI), also known as chorioamnionitis, is an infection of the amniotic fluid, placenta, fetal membranes, fetus, and/or decidua. One should suspect IAI, as per the American College of Obstetricians and Gynecologists Committee opinion on “Intrapartum Management of Intraamniotic Infection,” when the mother’s temperature is *>*39.0°C or when it is 38.0°C–38.9°C in the presence of another clinical risk factor such as maternal leukocytosis, purulent cervical drainage, or fetal tachycardia.^1^ Infants born to mothers with IAI commonly receive antibiotic treatment as per the 2010 Centers for Disease Control and Prevention and the 2012 American Academy of Pediatrics (AAP) guidelines for early-onset bacterial sepsis evaluation (EOS).^2,3^ Maternal signs and symptoms suggestive of IAI were seen as a significant risk factor for EOS.^3,4^ More recently, neonatal literature has suggested a change of practice regarding these infants’ evaluation and treatment.^5–14^ Jan et al reviewed various management strategies of asymptomatic infants born to mothers with IAI.^6^ They highlighted the benefits and minimal risk of less antibiotic exposure, including promoting maternal-infant bonding and breastfeeding, decreased overall health care costs, and decreased length of stay.^6^ Additional authors have since stated concerns regarding antibiotic exposure, including altering the microbiome and highlighting that well-appearing, asymptomatic infants are unlikely to have sepsis.^7,8^

The updated AAP guideline published in 2018 for Early-Onset Bacterial Sepsis in Neonates *>* 35 0/7 weeks^10^ noted the substantial decline in EOS over the last few decades. It recommended alternative diagnosis and treatment pathways of these infants that include risk stratification. Risk stratification methods include categorical risk assessment, multivariate risk assessment, and risk assessment based primarily on the newborn clinical condition. Categorical risk assessment focuses on intrapartum risk factor threshold values as markers of increased risk for EOS, such as GBS-specific algorithms. Multivariate risk assessment uses selected intrapartum risk factors with the newborn clinical condition to estimate the infant’s risk of EOS. One example of this is the Kaiser Permanente early-onset sepsis calculator.^11^ Risk assessment primarily based on newborn clinical condition relies on clinical signs of illness to identify EOS. A quality improvement project at Lucile Packard Children’s Hospital Stanford focused more on risk assessment based on clinical presentation than on labs to guide the treatment of infants born to mothers with IAI.^12,13^ Some centers have used a combination of the above with laboratory tests.^10^ The new AAP guideline asks birth centers to consider developing a locally tailored, documented guideline for EOS risk assessment and clinical management with recommended ongoing surveillance of the guideline. We created and implemented a new guideline for asymptomatic infants born to mothers with IAI in our unit based on categorical risk assessment in singling out IAI and combined it with laboratory tests and serial newborn clinical examinations.

We aimed to decrease the antibiotic exposure from 100% to 20% for asymptomatic infants born to mothers with IAI by July 31, 2019.

## METHODS

*Context*: We conducted this project in a 13 bed Community Level 3 neonatal intensive care unit (NICU) located in a delivery hospital that, on average, delivers 3100 infants and admits 350 infants to the NICU per year. Neonatologists cover the NICU, and pediatricians cover couplet care. Historically, treatment of infants *older than 35 weeks* born to mothers with IAI required admission to the NICU and antibiotics for an average of 55 patients per year. We obtained baseline data on the treatment of these infants from January 2018 to January 2019.

**Fig. 1.**
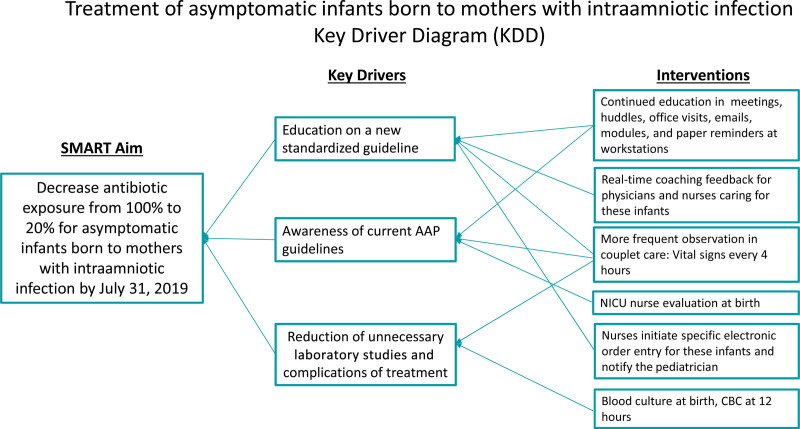
Key Driver Diagram.

**Fig. 2.**
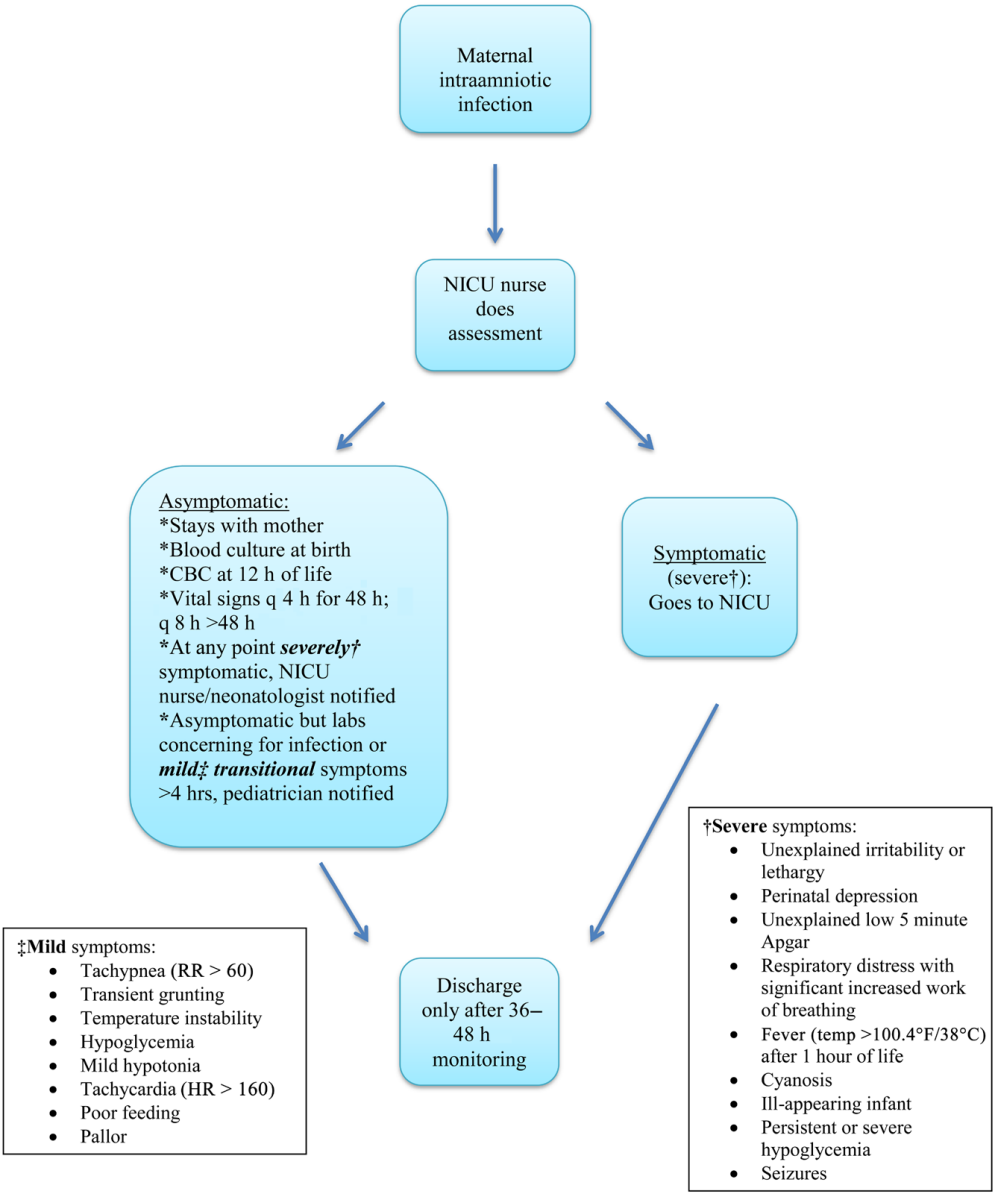
New standardized guideline for the treatment of asymptomatic infants born to mothers with intraamniotic infection.

*Interventions:* At the start of our QI effort, we formed a multidisciplinary team composed of physicians and nurses from obstetrics, neonatology, and pediatrics. The aims were determined from the baseline data. We created a key driver diagram (Fig. 1) to identify and address critical issues and interventions. The previous policy was to admit all infants born to mothers with IAI to the NICU for EOS evaluation with antibiotic use. The obstetric team diagnosed IAI for the mothers before, during, or just after delivery of the infant. Infants born to mothers diagnosed with IAI were admitted to the NICU for peripheral intravenous line placement, evaluation with complete blood count (CBC) and blood culture, and administration of 48 hours of antibiotics. The team discussed alternative diagnosis and treatment pathways for these infants and made recommendations based on current NICU management, the updated AAP guideline,^10^ and a literature review. A new standardized guideline for the treatment of infants born to mothers with IAI was reviewed and modified before its implementation (Fig. 2).

As per the new standardized guideline, infants are evaluated at birth by the NICU nurse in the delivery room. Asymptomatic infants remain with their mothers, and monitoring occurs without the initiation of antibiotics. The newborn nurse admits these infants under specific order sets in communication with the labor and delivery and postpartum nurses. This intervention was a change in the transition of care for infants from labor and delivery to the postpartum area as per the new guideline. They receive a blood culture at birth, CBC at 12 hours, and clinical evaluations by postpartum nurses with more frequent vital signs every 4 hours instead of the standard every 8 hours. Postpartum nurses cover up to 4 mother/infant dyads per shift. Pediatricians perform a comprehensive physical examination daily. Infants with mild transitional symptoms, such as tachypnea, grunting, temperature instability, or poor feeding, that last > 4 hours or laboratories of concern to the pediatrician for infection, including increased or decreased white blood cell count and/or bandemia, are further evaluated by the pediatrician. He/she can then notify the neonatologist for further evaluation and consideration for NICU admission with possible antibiotic treatment. At any point, if the infant has a positive blood culture and/or exhibits severe symptoms, such as unexplained irritability or lethargy, or significantly increased work of breathing, providers admit them to the NICU for observation and treatment with antibiotics. Pediatricians discharge infants from couplet care only after 36–48 hours of monitoring.

*Implementation:* Education for >200 pediatricians, obstetricians, and newborn and NICU nursing staff occurred at department meetings, monthly nurse staff meetings, nursing huddles, office visits, and via multiple emails over 3 months. Nursing leaders and neonatologists distributed the new guideline to staff in various forms—from email to flyers to laminated copies by the pediatrician staff desk. The obstetric team was reeducated on American College of Obstetricians and Gynecologists’s IAI diagnostic criteria.^1^ The multidisciplinary team met monthly to review new data, evaluate interventions, and discuss strategies for change. PDSA cycles related to deviations from the guideline were then used to make necessary changes. We continued education with real-time coaching feedback for all nurses and physicians caring for these infants, including the women’s services educator and the neonatologist personally talking with individuals who veered from the guideline.

*Measures:* The outcome measure was the antibiotic exposure of asymptomatic infants born to mothers with IAI. The process measures were the NICU admission rate and the length of hospital stay for asymptomatic infants born to mothers with IAI. Compliance with the guideline would lead to decreased NICU admission rate and limit the prolonged length of hospital stay if the infant remained asymptomatic without concerning laboratories or a positive blood culture. The balancing measure was readmission of asymptomatic infants born to mothers with IAI for infection within 30 days of life.

The delivery hospital’s quality team sent monthly updates on the number of mothers diagnosed with IAI in the past month. The team reviewed the mothers’ and infants’ charts to confirm the diagnosis and gather data. Data gathered on the infant included: date of birth, discharge date, length of stay, any NICU admission, any readmissions in 30 days, sex, gestational age, blood culture results, CBC timing and results, maternal name, presence of NICU nurse at delivery, frequency of vital signs during the stay, and infant and maternal clinical courses. Exclusion criteria included any symptomatic infant born to a mother with IAI admitted to the NICU or any infant readmitted for noninfectious related reasons.

*Data analysis:* We collected baseline data on the treatment of infants born to mothers with IAI for 13 months before the intervention (January 2018–January 2019). With the implementation of the new standardized guideline, data were collected monthly from February 2019 to December 2019. Quarterly audits commenced in January 2020. Monthly data analysis was collected from February 2019–December 2019 to allow rapid changes to the current process. Run charts and control charts allowed for descriptive analysis of the data.^15^ Basic statistics were performed by calculating the mean and median of the processes analyzed. A shift in the centerline was greater than or equal to 8 consecutive points, either above or below the average. Chi-square test and unpaired, 2-sample *t* test with unequal variance compared our categorical and continuous data, respectively. We examined the relationship between implementing changes in care with changes in the process and outcome measures. We used annotated statistical process control charts to establish baselines and track progress over time.

*Ethical Considerations:* The local institutional review board determined that the project was not human subjects research, and it was exempt from review.

## RESULTS

**Fig. 3.**
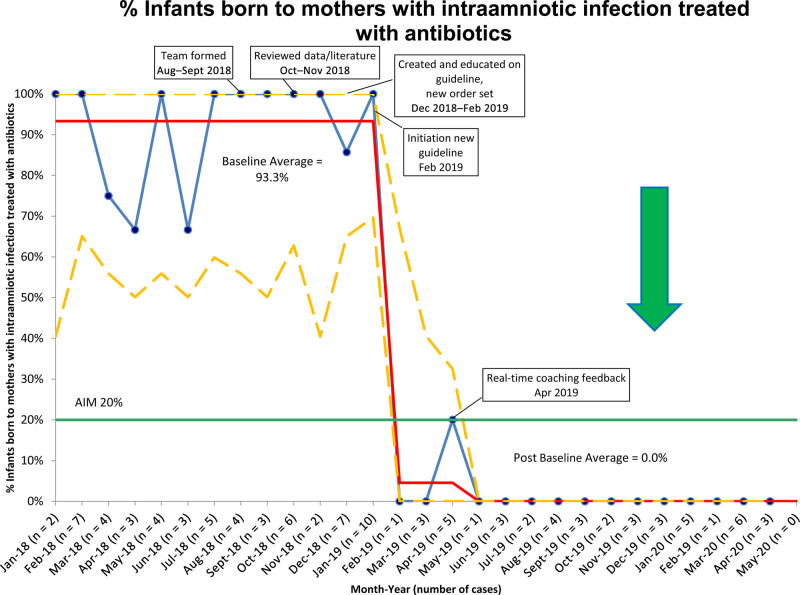
P-chart with monthly data on % of infants born to mothers with intraamniotic infection treated with antibiotics from pre- and post-implementation of the new guideline.

Baseline data revealed that we admitted 56/60 (93%) asymptomatic infants born to mothers with IAI over 13 months to the NICU and treated them with at least 48 hours of antibiotics with an average length of stay of 3.8 days. After implementing the new guideline, we admitted 1/45 (2%) asymptomatic infants born to mothers with IAI to the NICU for evaluation and treatment with antibiotics (Fig. 3). The remaining infants stayed with their mothers with an average length of stay of 3.2 days (*P* = 0.0668). There were no positive blood cultures and no known readmissions for infection within 30 days of discharge. Continued data review shows that no asymptomatic infants born to mothers with IAI were admitted to the NICU to evaluate and treat IAI since April 2019. The number of asymptomatic infants born to mothers diagnosed with IAI was higher in the baseline period, 60 infants/3375 live births (1.8%), than post-implementation of the new guideline, 45 infants/4087 live births (1.1%; *P* = 0.014, significant at *P* < 0.05). The number of symptomatic infants was the same between both periods (8 infants for each, respectively). Although a slight difference was noted, the total number of live births was similar between 2018 (3112) and 2019 (3116). Additionally, recent numbers from 2020 (1 year after implementing the guideline and initial education on IAI) show that the overall IAI diagnosis rate is slightly increased (15 infants/1234 live births = 1.2%).

## DISCUSSION

Treatment of asymptomatic infants born to mothers with IAI may expose these infants to unnecessary antibiotics while increasing risks, such as altering the microbiome, decreasing maternal-infant bonding, and altering breastfeeding initiation.^5–14^ Recent literature and the 2018 AAP guideline show the decline in EOS and consideration for the use of risk stratification or assessment.^7–11^ Numerous institutions utilize the EOS calculator, which has helped stratify risk for various infants, including those born to mothers with IAI, to decrease antibiotic exposure.^11,16–20^ This falls under multivariate risk assessment as per the new AAP guideline and as described above in the introduction. Our project used the new AAP guideline’s categorical risk assessment in singling out IAI. We combined it with laboratory tests and serial newborn clinical examinations to highlight to medical and nursing staff that asymptomatic infants born to mothers with IAI can remain monitored in couplet care.

Quality improvement is dependent on the education of all those involved and a strong multidisciplinary team that supports the initiative.^20–23^ Education of our team before and during this project and continuous communication were critical to our success. Having support from the NICU nurse and/or neonatologist for any questions the postpartum nurse and/or pediatrician had was essential. During the monthly reviews of cases, it was notable that the most common deviations from the guideline included lapses in frequent vital signs and clinical checks (most common after the first 24 hours), not notifying the NICU nurse or the pediatrician as to the maternal diagnosis of IAI (limiting or delaying the evaluation of the infant), and the timing of CBCs (done at birth or shortly after versus at 12 hours). Continuing education and real-time coaching feedback for physicians and nurses caring for these infants helped decrease the guideline deviations. The neonatologist reeducated 2 physicians on the guideline in the first few months. The physicians then followed the guideline with subsequent cases.

A few cases since the implementation of this project occurred in which infants born to mothers coded with IAI did not receive blood cultures and CBCs. Two cases involved low-grade maternal intrapartum fevers in which pediatricians were aware of the fever but did not realize the official diagnosis of IAI. Three cases involved maternal postpartum fever and a delayed diagnosis of IAI in which pediatricians were not aware of or updated as to the diagnosis. Vital signs were done more frequently in most of these infants, suggesting that nurses were aware of the maternal diagnosis but did not follow the guideline. Alternatively, the pediatrician may have known the diagnosis, but given the delay in diagnosis, chose not to follow the guideline knowing the infant clinically was doing well. Similarly, 4 infants from baseline data were not admitted to the NICU for antibiotic treatment and monitored in couplet care for maternal fever, later diagnosed by the obstetric team as IAI.

These diagnostic delays and labeling of maternal fever versus endometritis versus IAI also highlight the nonspecific signs and symptoms used to diagnose IAI and the diagnostic dilemma this creates.^1,6,8,10,11,14,24^ Ideally, better communication between the obstetrician, postpartum nurse, and pediatrician would have prompted more complete evaluations in the infants above, which highlights the need for continued education and real-time coaching feedback. Ultimately, all of these infants were discharged with their mothers with no known readmissions for infection within 30 days of discharge.

An interesting finding was the lower numbers of IAI diagnoses in asymptomatic infants after implementing this project. The team predicted that the number of IAI diagnoses would have increased given the recent education and spotlight on IAI. It did not! We postulate that increased awareness of the defined American College of Obstetricians and Gynecologists criteria for suspected IAI infections in the first year led to fewer diagnosed cases. Recent numbers from 2020 show an upward trend in diagnoses. Overall delivery numbers have remained similar to previous years during the implementation of the project. It would be interesting to trend the IAI diagnosis rate with reeducation on IAI.

Limitations in this study were the low numbers of asymptomatic infants born to mothers with IAI, the low incidence of EOS as noted in the 2018 updated AAP guideline,^10^ the potential readmission of infants outside our catchment area, and the variability in diagnosing IAI among the obstetric team. Although the decline in antibiotic exposure may not have been as dramatic without these limitations, we believe these are limitations shared by many community hospitals. Our new guideline led to a shift in antibiotic use with minimal adverse effects that can be applied in similar community hospital situations.

The next steps for the project would be to include more intrapartum risk factors with frequent newborn clinical monitoring to identify infants at risk for EOS.

## CONCLUDING SUMMARY

Implementing best antibiotic stewardship practices through a standardized guideline and education by a multidisciplinary team to gain staff support led to a decline in antibiotic exposure and NICU admission for asymptomatic infants born to mothers with IAI. There was no known increase in readmissions for infection but decreased overall IAI diagnoses. Continued education on this guideline with regular audits has maintained the benefits gained. More significant numbers or additional studies could help validate these guidelines.

## DISCLOSURE

The authors have no financial interest to declare in relation to the content of this article.

## ACKNOWLEDGMENTS

Kathleen Diamond, MSN, our wonderful NICU nurse champion and educator for this project, Gina Lowery, MSN, and Cynthia Anthony, BSN, director and manager of Women’s Services at Rancho Springs, respectively, were instrumental in disseminating this project in Women’s Services.

## References

[1] Heine RP, Puopolo KM, Beigi R, et al; Committee on Obstetric Practice. Committee Opinion No. 712: Intrapartum management of intraamniotic infection. Obstet Gynecol. 2017;130:e95–e101. 28742677 10.1097/AOG.0000000000002236

[2] Verani JR McGee L Schrag SJ; Division of Bacterial Diseases, National Center for Immunization and Respiratory Diseases, Centers for Disease Control and Prevention (CDC). Prevention of perinatal group B streptococcal disease–revised guidelines from CDC, 2010. MMWR Recomm Rep. 2010;59(RR-10):1–36. 21088663

[3] Polin RA; Committee on Fetus and Newborn. Management of neonates with suspected or proven early-onset bacterial sepsis. Pediatrics. 2012;129:1006–1015. 22547779 10.1542/peds.2012-0541

[4] Malloy MH. Chorioamnionitis: epidemiology of newborn management and outcome United States 2008. J Perinatol. 2014;34:611–615. 24786381 10.1038/jp.2014.81

[5] Wortham JM Hansen NI Schrag SJ. Chorioamnionitis and culture-confirmed, early-onset neonatal infections. Pediatrics. 2016;137:e20152323. 10.1542/peds.2015-2323 PMC4702021 26719293

[6] Jan AI Ramanathan R Cayabyab RG. Chorioamnionitis and management of asymptomatic infants ≥35 weeks without empiric antibiotics. Pediatrics. 2017;140:e20162744. 28759393 10.1542/peds.2016-2744

[7] First L. Is it time to rethink our management of chorioamnionitis in an otherwise asymptomatic late preterm or term infant? Pediatrics . Journals Blog. June 8, 2017. Available at https://www.aappublications.org/news/2017/06/08/Is-It-Time-To-Rethink-Our-Management-Of-Chorioamnionitis-In-An-Otherwise-Asymptomatic-Late-Preterm-Or-Term-Infant-Pediatrics-6-8-17?utm_source=TrendMD&utm_medium=TrendMD&utm_campaign=AAPNews_TrendMD_0. Accessed February 15, 2019.

[8] Hooven TA Polin RA. Time to overhaul the “Rule Out Sepsis” workup. Pediatrics. 2017;140:e20171155. 28759417 10.1542/peds.2017-1155

[9] Randis TM Polin RA Saade G. Chorioamnionitis: time for a new approach. Curr Opin Pediatr. 2017;29:159–164. 28134708 10.1097/MOP.0000000000000466

[10] Puopolo KM Benitz WE Zaoutis TE; Committee on Fetus and Newborn; Committee on Infectious Diseases. Management of neonates born at ≥35 0/7 weeks’ Gestation with suspected or proven early-onset bacterial sepsis. Pediatrics. 2018;142:e20182894. 30455342 10.1542/peds.2018-2894

[11] Kuzniewicz MW Puopolo KM Fischer A. A quantitative, risk-based approach to the management of neonatal early-onset sepsis. JAMA Pediatr. 2017;171:365–371. 28241253 10.1001/jamapediatrics.2016.4678

[12] Joshi NS Gupta A Allan JM. Clinical monitoring of well-appearing infants born to mothers with chorioamnionitis. Pediatrics. 2018;141:e20172056. 29599112 10.1542/peds.2017-2056

[13] Joshi NS Gupta A Allan JM. Management of chorioamnionitis-exposed infants in the newborn nursery using a clinical examination-based approach. Hosp Pediatr. 2019;9:227–233. 30833294 10.1542/hpeds.2018-0201

[14] Chiruvolu A Petrey B Stanzo KC. An institutional approach to the management of asymptomatic chorioamnionitis-exposed infants born ≥35 weeks gestation. Pediatr Qual Saf. 2019;4:e238. 32010864 10.1097/pq9.0000000000000238 PMC6946240

[15] Benneyan JC Lloyd RC Plsek PE. Statistical process control as a tool for research and healthcare improvement. Qual Saf Health Care. 2003;12:458–464. 14645763 10.1136/qhc.12.6.458 PMC1758030

[16] Kerste M Corver J Sonnevelt MC. Application of sepsis calculator in newborns with suspected infection. J Matern Fetal Neonatal Med. 2016;29:3860–3865. 26948457 10.3109/14767058.2016.1149563

[17] Warren S Garcia M Hankins C. Impact of neonatal early-onset sepsis calculator on antibiotic use within two tertiary healthcare centers. J Perinatol. 2017;37:394–397. 28005063 10.1038/jp.2016.236

[18] Achten NB Dorigo-Zetsma JW van der Linden PD. Sepsis calculator implementation reduces empiric antibiotics for suspected early-onset sepsis. Eur J Pediatr. 2018;177:741–746. 29455368 10.1007/s00431-018-3113-2

[19] Strunk T Buchiboyina A Sharp M. Implementation of the neonatal sepsis calculator in an Australian tertiary perinatal centre. Neonatology. 2018;113:379–382. 29514161 10.1159/000487298

[20] Akangire G Simpson E Weiner J. Implementation of the neonatal sepsis calculator in early-onset sepsis and maternal chorioamnionitis. Adv Neonatal Care. 2020;20:25–32. 31569094 10.1097/ANC.0000000000000668

[21] Horbar JD Plesk PE Leahy K; NIC/Q 2000: NIC/Q 2000: Establishing habits for improvement in neonatal intensive care units. Pediatrics. 2003;111(4 Pt 2):e397–e410. 12671159

[22] Horbar JD Rogowski J Plsek PE. Collaborative quality improvement for neonatal intensive care. NIC/Q Project Investigators of the Vermont Oxford Network. Pediatrics. 2001;107:14–22. 11134428 10.1542/peds.107.1.14

[23] Langley GL Moen RD Nolan KM. The Improvement Guide: A *Practical Approach* to *Enhancing Organizational Performance*. 2nd ed. Jossey-Bass; 2009.

[24] Greenberg MB Anderson BL Schulkin J. A first look at chorioamnionitis management practice variation among US obstetricians. Infect Dis Obstet Gynecol. 2012;2012:628362. 23319852 10.1155/2012/628362 PMC3540735

